# Robust CRISPR/Cpf1 (Cas12a)‐mediated genome editing in allotetraploid cotton (*Gossypium hirsutum*)

**DOI:** 10.1111/pbi.13147

**Published:** 2019-06-03

**Authors:** Bo Li, Hangping Rui, Yajun Li, Qiongqiong Wang, Muna Alariqi, Lei Qin, Lin Sun, Xiao Ding, Fuqiu Wang, Jiawei Zou, Yanqing Wang, Daojun Yuan, Xianlong Zhang, Shuangxia Jin

**Affiliations:** ^1^ National Key Laboratory of Crop Genetic Improvement Huazhong Agricultural University Wuhan Hubei China; ^2^ Institute of Nuclear and Biological Technologies Xinjiang Academy of Agricultural Sciences Urumqi Xinjiang China; ^3^ Xinjiang Production and Construction Corps Key Laboratory of Protection and Utilization of Biological Resources in Tarim Basin Tarim University Alaer Xinjiang China

**Keywords:** allotetraploid cotton, genome editing, CRISPR/Cpf1 (Cas12a), off‐target

As an allotetraploid, most genes have multiple copies that belong to At and Dt subgenomes in cotton (*Gossypium hirsutum*). Allotetraploid cotton genome is very complex (AADD, 2n = 4x = 52) with a large genome size of 2.5 Gb. As a result, different types of genome editing tools are desirable to improve the functional genomic research in cotton. CRISPR/Cpf1 (Cas12a) is a novel member of the CRISPR/Cas system from *Alicyclobacillus acidoterrestris*, and currently, three kinds of Cpf1 are commonly used in genome editing, namely AsCpf1, LbCpf1 and FnCpf1. In 2015, type V CRISPR/Cpf1 (Cas12a) was applied in human cells to create gene knockout mutation for the first time (Zetsche *et al*., 2015). Several applications in rice showed that LbCpf1 exhibited higher genome editing efficiency than AsCpf1 and spCas9 (Tang *et al*., 2017, 2018). The LbCpf1 used in this study is from *Lachnospiraceae bacterium* ND2006 (LbCpf1, NCBI accession number: NZ_JNKS01000011; gene locus_tag: T521_RS08385) consisting of 1228 amino acids (Zhong *et al*., 2018). Unlike the Cas9 requiring higher G/C content in PAM sites, the PAM sequence of Cpf1 is 5′‐TTTV‐3′, which prefers genes with higher A/T content. The crRNA of Cpf1 is 23 nt in length (the PAM sequence is upstream of the target sites). The editing process of Cpf1 does not require the participation of tracrRNA, and the pre‐crRNA can be self‐modified into a mature crRNA. The Cpf1 binds to the mature crRNA to form a binary complex, and then, this complex binds to the target DNA to form a ternary complex (Xu *et al*., 2018). Recently, several groups have successfully applied the CRISPR/Cpf1 system in plant species such as rice, soya bean, tobacco and maize (Lee *et al*., 2019; Tang *et al*., 2017, 2019). Notably, no off‐target mutations were detected at the potential off‐target sites in the LbCpf1‐edited rice plant, suggesting that LbCpf1 is a robust and precise genome editing tool after the Cas9 system (Tang *et al*., 2018). However, this promising system has not been tested in cotton yet. Here, we established an efficient CRISPR/LbCpf1 system to expand the scope of genome editing in allotetraploid cotton for the first time.

To test the efficiency of LbCpf1 system in cotton, we constructed an LbCpf1 plasmid vector that harbors 23‐nt crRNA to target the cotton endogenous gene *cloroplastos alterados* (*GhCLA*). A previous work has shown that tRNA‐sgRNA transcription unit is very effective to enhance the sgRNA transcription in CRISPR/Cas9 system for cotton genome editing (Wang *et al*., 2018). Therefore, we designed the crRNA following our previous work and conducted a vector named pGhRBE3‐*Cpf*1‐*GhCLA*1 targeting *GhCLA*1 gene (Figure 1a). Several independent regenerated plants were obtained through *Agrobacterium*‐mediated transformation following our previous protocol (Wang *et al*., 2018). In total, 92 independent regenerated plants were obtained in the T0 generation after the genetic transformation containing LbCpf1 plasmid vector. Mutations in the generated transgenic plants were detected by high‐throughput (Hi‐Tom) sequencing and Sanger sequencing (Liu *et al*., 2019). The targeted region of *GhCLA*1 was amplified by PCR using site‐specific primers and used for mutation detection. The PCR products of the 92 T0 plants containing the target sites were pooled and purified to conduct high‐throughput sequencing, whereas Sanger sequencing results showed that 80 out of 92 (87% efficiency) independent T0 plants were detected with mutations at the target site, suggesting the robust editing activity of LbCpf1 in cotton plants. On the other hand, Hi‐TOM data (original data have been submitted to http://gsa.big.ac.cn/) revealed that each individual plant contained diverse editing extent. Samples with target mutation ratio (the reads with target mutations/total reads of the target site) lower than 1% were considered as negative (no edition at the target site), whereas those samples with mutation ratio > 1% accounted as mutated in which mutations truly occurred at the target site. Based on this criterion, 12 individuals of the 92 independent plants were negative without any obvious targeted mutations, which are completely consistent with the Sanger sequencing data. The remaining 80 individuals were edited with mutation extent ranging from 1% to 94.12% (Figure 1b). Those plants (8 out of 80 T0 plants) with higher target mutations ratio (around 80% mutation ratio revealed by Hi‐TOM, indicated with red arrow in Figure 1b) exhibited homozygous phenotype (albino seedlings), while the plantlets with <80% mutation ratio were chimeric mutants with white spots in the leaves and the stems. The editing extent per plant was mainly concentrated in 20%–60% (Figure 1b). In summary, the CRISPR/Cpf1 system has high gene editing efficiency in cotton. Most of the generated T0 plants have been mutated at the target site. The editing window and profiling of CRISPR/Cpf1 system in the edited T0 plants were systematically analysed in this report. Since no target mutation occurs preceding the PAM sequence and within the PAM sequence, we investigated the editing downstream of the PAM sequence. The main editing window of CRISPR/Cpf1 ranged between the +13 and +25 downstream of the PAM sequence (Figure 1c). Comparing with base substitution and insertion, the CRISPR/Cpf1 system prefers to induce DNA deletion at the target sites rather than base substitution or insertion. Only deletions were detected at the crRNA target sites in all the 80 T0 plants. Among them, 68 samples were edited in At and Dt subgenomes simultaneously, and the rest of the samples were edited only in At or Dt subgenome (Figure 1d). Deletion size and proportion of each target mutation in T0 plants were also analysed and are shown in Figure 1e. The deletion sizes ranged from 3 to 28 bp in length, and the deletion size of majority is from 5 to 12 bp (Figure 1e), which are larger than the average deletion size (1–5 bp) induced by CRISPR/Cas9 in cotton genome (Wang *et al*., 2018). Therefore, we speculate that the CRISPR/Cpf1 system prefers to generate large size deletions in cotton genome editing.

**Figure 1:**
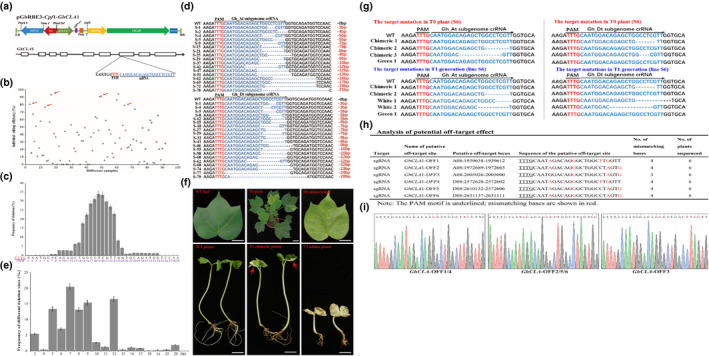
CRISPR/Cpf1 (Cas12a)‐mediated genome editing in cotton. (a) T‐DNA region of the pGhRBE3‐Cpf1 vector for target mutation in cotton. The crRNA is located on the seventh exon of the *GhCLA1*. (b) Target mutation extent of T0 plants edited by cotton CRISPR/Cpf1 (Cas12a) system. The mutation frequency of the *GhCLA* 1 gene in the independent T0 plant (revealed by Hi‐TOM) ranged from 1% to 94.12%, and the editing extent per plant was mainly concentrated in 20%–60%. Those plants with higher target mutations ratio (around 80% mutation ratio indicated with red arrow) exhibited nearly homozygous phenotype (nearly albino seedlings). The target mutation efficiency of CRISPR/Cpf1 system in cotton reached 87% (80 out of 92 T0 plants were detected with target mutations by Sanger sequencing and Hi‐TOM analysis). (c) Frequencies of DNA deletion at the target sites of *GhCLA1*. The deletion mainly occurs between the 13th and 18th nucleotide downstream of the PAM site. The PAM sites are highlighted in red. (d) The target mutation profiles of T0 plants edited by cotton CRISPR/Cpf1 (Cas12a) system. Only DNA deletions were detected at the target sites of all the T0 plants. (e) Frequency of different deletion sizes at the target sites of *GhCLA1*. The shortest DNA deletion is 3 bp, and the longest one is 28 bp. The deletion size is mainly between 5 and 12 bp measured by high‐throughput (Hi‐Tom) sequencing; bp: base pairs. (f) Phenotype of the T0 and T1 cotton plants with the target mutations in *GhCLA1* gene. T0 plant (S6) exhibited chimeric phenotype with white spots scattered in green leaves. Some T1 progenies (Line S6) from chimeric T0 plant (S6) are homozygous with completely albino seedlings, and some T1 plants still show chimeric phenotype just like their parental T0 plant with white spots (indicated with the arrow) scattered in green leaves. Bar = 1 cm. (g) Genotype revealed by Sanger sequencing from T0 (S6) to T1 generation cotton plants. The target mutation profile in the crRNA target sites of T1 plants was similar to that of T0 parental plants. Chimeric‐1 and 2: the samples from a whole piece of leaf with white and green parts; White‐1: a full white leave piece and full green parts (Green‐1). And all these samples were taken from the same T0 plant (S6). The PAM sites are highlighted in red, and crRNA sequence sites are highlighted in blue. (h, i) Analysis of potential off‐target effect in T1 plants (Line S6). Off‐target effects were detected at 6 predicted potential off‐target sites in 6 independent T1 plants by Sanger sequencing. The PAM motif is underlined; mismatching bases are shown in red.

Generally, *GhCLA1*‐mutated plants (homozygous mutants) should be albino if the *GhCLA1* gene was completely knocked out. However, in this report, all the analysed T0 plants exhibited chimeric phenotype (with white spots in the leaves and stems) (Figure 1f, upper panel) because the homozygous mutants of *GhCLA1* cannot survive in the soil. The chimeric phenotype in the T0 generation was also very common in the CRISPR/Cas9‐edited cotton plants due to the gene redundancy. Each gene in the allotetraploid cotton genome has almost four loci at At and Dt subgenomes, and to obtain the homozygous mutants, all the gene loci should be edited simultaneously (Wang *et al*., 2018). For further determination of whether the target sites of the *GhCLA1* gene in the heterozygous plants were edited or not, the following three types of leave samples were analysed: a whole piece of leaf with white and green parts (Chimeric‐1 and 2), a full white leaf piece (White‐1) and full green leaf piece (Green‐1), and all these samples were taken from the same T0 plant (S6). The Sanger sequencing data showed that only one copy of *GhCLA1* at At or Dt subgenome was mutated in Chimeric‐1 and 2 samples and in the White‐1 sample, and all the loci at At and Dt subgenomes gained the target mutations. Obviously, there are no target mutations in At nor Dt subgenomes of the Green‐1 sample (Figure 1g, upper panel). To detect the inheritance of the editing from T0 to T1 generation, the phenotype of T1 progeny from 4 chimeric T0 plants was recorded and all these four T1 lines exhibited inherited mutations at the target sites. As shown in line S6, some of the progeny were homozygous with completely albino seedlings and all the loci in the At and Dt subgenomes were mutated simultaneously, but their growth was significantly stunted. Notably, almost half of T1 plants from line S6 still showed chimeric phenotype just like their parental T0 plants with white spots scattered in the green leaves. The Sanger sequencing data reveal the same genotype in T1 progeny (White‐1 and White‐2) as the chimeric samples (Chimeric‐1 and 2) from the T0 parental plant (S6) with partial mutation in the At or Dt subgenome, and no target mutation was detected in the green T1 plant (sample Green‐1) (Figure 1f, lower panel). The other three T1 lines showed similar phenotypes with line S6 (some plants are homozygous and others are chimeric). Depending on the genotype profiling of T1 plants, most editing types in T0 plants were faithfully inherited to all the T1 progenies (Albino plants, chimeric plants and green plants) and confirmed by Sanger sequencing (Figure 1g, lower panel). In addition to the on‐target editing of the CRISPR/Cpf1 in cotton, the off‐target effects were also investigated in this report. We identified the most six potential off‐target sites based on the method described in sgRNAcas9_3.0.5 software and our recent report (Xie *et al*., 2014). These potential off‐target sites of T1 plants were tasted by Sanger sequencing using their site‐specific PCR products. Sequencing results showed that no off‐target effects were detected in all the predicted off‐target sites (Figure 1h–i), concluding that CRISPR/Cpf1 system is highly specific and can be a good option for cotton genome editing.

Overall, in this study, we successfully built a site genome editing in allotetraploid cotton with 87% editing efficiency and no off‐target effects using CRISPR/Cpf1 system. Our results are comparable with the editing efficiency in rice and maize (Lee *et al*., 2019; Tang *et al*., 2017; Xu *et al*., 2018). More importantly, the phenotypic and the genetic edition occurred in the T0 generation were inherited faithfully to their progenies and some homozygous mutants were obtained in the T1 generation. In the future, whole‐genome sequencing (WGS) will be applied to evaluate the off‐target of CRISPR/Cpf1 system in cotton. These results support the finding that the CRISPR/Cpf1 system is a highly specific and efficient system in plant genome editing, which will be a very promising alternative of the CRISPR/Cas9 system in cotton.

## Conflict of interest

The authors declare no conflicts of interest.
